# Skin irritation in children undergoing orthodontic facemask therapy

**DOI:** 10.1038/s41598-023-29253-0

**Published:** 2023-02-07

**Authors:** Harim Kim, Jung Suk Kim, Cheol Soon Kim, Su Youn Becker-Weimann, Jung-Yul Cha, Sung-Hwan Choi

**Affiliations:** 1 Department of Orthodontics, Institute of Craniofacial Deformity, Yonsei University College of Dentistry, 50-1 Yonsei-ro, Seodaemun-gu, 03722 Seoul, Republic of Korea; 2 Private Clinic, Seongnam-si, Republic of Korea; 3 Department of Dermatology, Venereology, Allergology, Goethe University, Frankfurt am Main, Germany

**Keywords:** Craniofacial orthodontics, Paediatric dentistry

## Abstract

Orthodontic facemasks are extraoral orthodontic appliances that influence maxillary and mandibular development in children with skeletal Class III malocclusion. While a facemask is most effective in patients before the growth spurt, skin irritation is common during the treatment. Therefore, this retrospective study aimed to investigate the prevalence and pattern of such skin changes and identify their possible associated risk factors. We included 177 patients with skeletal Class III malocclusion who underwent facemask therapy. Patient age and sex, orthodontic parameters expressing the severity of malocclusion, the presence of complaints in the temporomandibular joint (TMJ) areas, and the level of patient cooperation were evaluated. Additionally, the severity and onset time of skin reactions were further analyzed. The results indicated that 43.5% of patients developed skin changes typical of irritant contact dermatitis. Skin irritation was significantly associated with the presence of TMJ complaints and female sex. Furthermore, skin irritation was more common in younger patients. Clinicians should pay special attention to the skin areas that come into contact with the appliance during each follow-up visit to detect potential problems. Moreover, patients and their parents should be given adequate information about the possibility, prevention, and management of skin problems during facemask therapy.

## Introduction

Orthodontic facemasks, known as protraction or reverse headgear, have been used for nearly 50 years to treat growing skeletal Class III patients^1^. Skeletal Class III malocclusion is characterized by maxillary hypoplasia, mandibular prognathism, or both, and can be effectively treated during growth by applying orthopedic forces to the jaw bones using a facemask. This treatment modality is effective in growing patients with patent sutures and is typically applied before the pubertal growth spurt to maximize the skeletal effects^2^. The orthopedic effects of facemasks are known to decrease drastically in postpubertal patients, and dentoalveolar rather than orthopedic effects are to be expected^3^. Delaire’s original 1971 design consists of a forehead support, a chin cup, two vertical bars on the sides connecting these two, and a crossbar for the application of elastics, which generate orthopedic forces^1,4^. Several changes to this design have been made to improve patient comfort^4–6^. In addition to the Delaire-type, the Petit-type facemask, which has a single central vertical bar instead of two, is now widely used. The forehead support and chin cup are essential components in both types, as they transmit reaction force to the forehead and chin areas. The forehead support and chip cup are supported by the forehead and chin, respectively, and come into direct contact with the facial skin when the appliance is worn. Therefore, these components may cause skin reactions in patients undergoing facemask therapy. Skin problems in the chin area, in particular, are frequently observed during facemask therapy and are widely accepted as one of the complications of facemask therapy. This is most likely due to the facemask’s appliance design, which exerts significantly more pressure on the chin than on the forehead^7^. Facemask, as a removable, compliance-dependent appliance, requires sufficient wearing time. A consistent application of 10–14 h per day for approximately 6–12 months is usually recommended for a successful treatment^8^. Discomfort or pain caused by skin irritation can negatively affect patient cooperation and wearing time, thereby delaying the success of facemask therapy. Despite widespread recognition of skin irritation caused by facemasks, there are few studies on this subject in the literature, and the potential causes have received little attention. This study aimed to investigate the occurrence of skin irritation during facemask therapy and determine the possible factors associated with it.

## Materials and methods

### Study population

We included 177 children with skeletal Class III malocclusion who were treated with a facemask at a private orthodontic clinic in Korea between January 2020 and June 2022. The inclusion criteria were as follows: (1) point A-Nasion-point B (ANB) < 0° and Wits appraisal < − 4 mm; (2) crossbite of at least one anterior tooth; (3) skeletal maturity indicators (SMI) obtained from hand-wrist radiographs indicating growth development before peak height velocity (PHV); and (4) absence of temporomandibular joint (TMJ) disorders. The exclusion criteria were as follows: (1) congenital craniofacial anomalies, including cleft lip and palate or syndromes affecting the craniofacial complex; (2) previous history of surgical intervention in the maxillofacial areas; and (3) insufficient quality of radiographs or the presence of foreign bodies affecting radiographic evaluation.

Depending on the patient’s skeletal pattern and the presence of deciduous teeth, a bonded rapid palatal expander (RPE) or a conventional RPE with hooks was used in conjunction with a facemask. The device was activated at a rate of 0.2 mm per day for 2 weeks. Following activation, suture separation was confirmed radiologically, and a Delaire-type facemask (Kwang Myung DAICOM Inc., Seoul, Korea) was delivered. From the two available sizes, the one that fits the patient’s face was chosen, and the vertical position of the forehead support and chin cup and the angulation of the chin cup were optimized for a better fit. The position of the crossbar and hooks for the application of elastics was adjusted to have a force directed approximately 30° downward from the occlusal plane with about 100–150 gm of protraction force on each side. Every month, the force was increased by 50–100 gm, eventually reaching 350–450 gm. One operator with over 25 years of experience in facemask therapy performed all the procedures. Patients were instructed to wear the appliance for at least 10 to 14 h daily, including at night.

The study was approved by the institutional review board of of Yonsei Dental Hospital (IRB No. 2-2022-0050) and adhered to the Declararion of Helsinki (2013). The requirement for written informed consent was waived considering the retrospective design of the study. All procedures, including radiography, were performed regardless of the study as part of routine care.

### Evaluation of skin irritation

**Figure 1:**
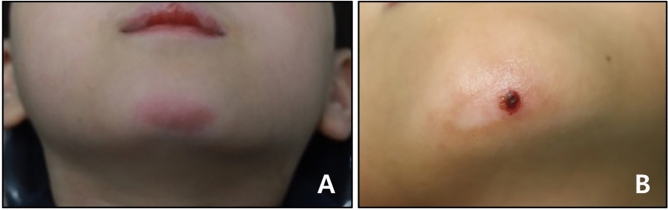
Skin irritation during facemask therapy. (**A**) Erythema (SII 1); (**B**) Laceration (SII 2).

Based on the patient records, the occurrence and severity of skin irritation observed during treatment were evaluated. The severity was determined using the skin irritation index (SII), with SII values of 0 indicating no skin change, 1 indicating redness, and 2 indicating skin disruption (Fig. 1)^9^. In patients who showed skin changes in the chin area, the onset of skin changes in the chin area was classified as occurring within 1 month, 2 months, or 3 months after the initiation of facemask therapy (Table 1).

**Table 1:** Skin irritation index and onset time of skin irritation.

Skin irritation index	Description	Onset time
0	No erythema (redness of skin) or laceration (disruption of skin)	–
1	Erythema	1 month 2 months 3 months
2	Laceration	

### Evaluation of malocclusion

**Figure 2:**
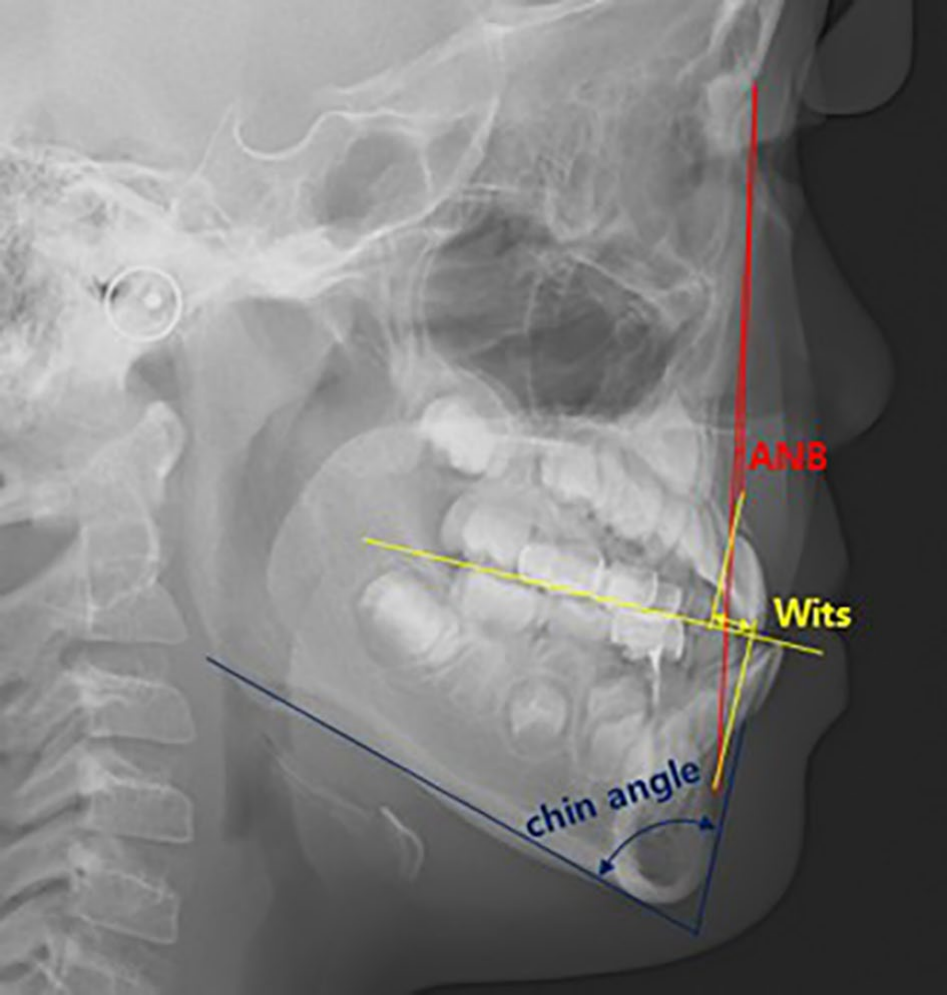
Cephalometric analysis used for the present study. ANB, point A-Nasion-point B angle; Wits appraisal, distance between the lines drawn from points A and B, perpendicular to the occlusal plane; chin angle, angle formed between the line passing through interdentale of lower incisors and pogonion and the mandibular plane.

Orthodontic parameters that indicate the severity of skeletal Class III malocclusion were assessed using pretreatment lateral cephalograms and dental casts. The parameters obtained from the cephalometric analysis included the ANB angle and Wits appraisal, and both assessed the anteroposterior relationship between the upper and lower jaw. Based on these results, the patients were classified into skeletal Class I, II, and III. Skeletal Class III is characterized by a decreased ANB angle and Wits appraisal, indicating the most pronounced anteroposterior discrepancy in favor of the mandible among the three skeletal groups (Fig. 2).

The anterior overjet and overbite were measured on pretreatment dental casts, and the patients were categorized as follows according to the results: overjet: (1) larger than 4 mm (increased overjet); (2) smaller than or equal to 4 mm and larger than 0 mm (normal overjet); (3) smaller than or equal to 0 mm (decreased overjet); and overbite: (1) larger than 4 mm (deep bite); (2) smaller than or equal to 4 mm and larger than 0 mm (normal overbite); or (3) smaller than or equal to 0 mm (open bite). Overjet reflects the horizontal distance between the upper and lower incisors and shows a negative value in patients with an anterior crossbite, which is again an indicator of excessive mandibular growth, a lack of maxillary growth, or a combination of both. Since the presence of an anterior crossbite was one of the inclusion criteria for the study, all patients had a negative overjet. Therefore, the overjet measured in this study was based on the rest of the anterior teeth, which were not in crossbite. A negative value was measured if all incisors were in crossbite.

### Evaluation of the chin angle

The chin angle was evaluated based on the pretreatment lateral cephalograms. It is measured as the angle between the mandibular plane and the chin line, which is formed by connecting the infradentale of the lower incisors and the pogonion. (Fig. 2)^10^. Because no known average value of chin angle was reported in the literature, the average value of the study population was used to categorize the results into groups above and below average for statistical analysis.

### Further variables

In addition to evaluating malocclusion and chin angle, the level of patient cooperation and the presence of TMJ complaints were assessed. The patients and their guardians were asked to report their daily wear time at each visit, which was recorded in patient charts. The level of patient cooperation was rated as good for patients who wore it for more than 12 h, fair for those who wore it for more than 10 h, and poor for those who wore it for less than 10 h. Patients who developed TMJ problems during treatment were identified using patient records. TMJ complaints included pain, restricted mouth opening, and clicking sounds.

### Statistical analysis

SPSS Statistics for Windows (version 22.0; IBM Corp., Armonk, NY, USA) was used to perform statistical analyses. A frequency analysis was performed, and the statistical significance of each variable for the occurrence, severity, and onset of skin irritation was tested using the chi-squared test. Fisher’s exact test was used if cells with counts of less than five were present. A binary logistic regression analysis was performed to further explore the effects of the study variables on the occurrence of skin irritation during facemask therapy. A *P*-value of < 0.05 was considered to be statistically significant.

## Results

Table 2 shows the demographic data of the study population. The sex distribution was slightly in favor of females, with 97 (54.8%) over 80 (45.2%) males. The age at the initiation of facemask therapy ranged from 5.58 to 13.5 years, and the mean age was 8.83 ± 1.376 years. The largest age group was 8–9 years, with 94 (53.1%) patients, while only 33 (18.6%) patients fell into the age group of over 10 years.

**Table 2:** Demographic data of the study population.

Demographic data		Frequency	%	Mean ± SD
Age (years)	6–7	50	28.2	8.83 ± 1.376
	8–9	94	53.1	
	> 10	33	18.6	
Gender	Female	97	54.8	
	Male	80	45.2	
Total		177	100.0	

Evaluation of the skin in the chin area revealed that 77 out of 177 patients experienced skin irritation during facemask therapy (Table 3). The proportion of patients with skin changes showed a decreasing trend with increasing age. 54% of the patients in the age group of 6–7 years experienced skin irritation, while in the age groups of 8–9 years and over 10 years, 40.4% and 36.4% showed skin problems, respectively. Furthermore, skin irritation occurred more frequently in female patients (50.5%) than in male patients (35%).

**Table 3:** The proportion of patients with skin irritation according to the variables.

Variable		Number of patients (percent)			x^2^ (*p*)
		Skin irritation	No skin change	Total	
Age (years)	6–7	27 (54.0%)	23 (46.0%)	50	3.288 (0.193)
	8–9	38 (40.4%)	56 (59.6%)	94	
	> 10	12 (36.4%)	21 (63.6%)	33	
Gender	Female	49 (50.5%)	48 (49.5%)	97	4.294 (0.038*)
	Male	28 (35.0%)	52 (65.0%)	80	
Overjet (mm)	0 < OJ ≤ 4	10 (31.3%)	22 (68.8%)	32	3.159^†^ (0.168)
	OJ > 4	0 (0.0%)	1 (100.0%)	1	
	OJ ≤ 0	67 (39.8%)	77 (60.2%)	144	
Overbite (mm)	0 < OB ≤ 4	51 (42.5%)	69 (57.5%)	120	0.162 (0.922)
	OB > 4	7 (46.7%)	8 (53.3%)	15	
	OB ≤ 0	19 (45.2%)	23 (54.8%)	42	
ANB	Class I	40 (38.8%)	63 (61.2%)	103	2.279 (0.320)
	Class II	7 (53.8%)	6 (46.2%)	13	
	Class III	30 (49.2%)	31 (50.8%)	61	
Wits	Class I	4 (36.4%)	7 (63.6%)	11	0.243^†^ (0.758)
	Class III	73 (44.0%)	93 (56.0%)	166	
TMJ complaints	Yes	10 (83.3%)	2 (16.7%)	12	8.309 (0.004*)
	No	67 (40.6%)	98 (59.4%)	165	
Patient cooperation	Good	18 (36.0%)	31 (64.0%)	50	4.121 (0.127)
	Fair	30 (40.5%)	44 (59.5%)	74	
	Poor	29 (54.7%)	24 (45.3%)	53	
Chin angle	> Average	45 (46.9%)	51 (53.1%)	96	0.971 (0.325)
	< Average	32 (39.5%)	49 (60.5%)	81	
Total		77 (43.5%)	100 (56.5%)	177 (100%)	

*p < .05.

^†^Fisher’s exact test.

Orthodontic parameters, including overjet, overbite, the ANB angle, and Wits appraisal, did not show any statistical significance for skin problems; however, some tendencies were observed. In the group with an overjet smaller than or equal to 0, 67 of 144 patients showed skin irritation (46.5%). In contrast, in the groups with normal overjet and increased overjet, the probabilities of skin irritation were 31.3% and 0%, respectively. Furthermore, regarding the anteroposterior relationship of the jaws as expressed by the ANB angle and Wits appraisal, patients with skeletal discrepancies showed a higher probability of skin irritation than those with a skeletal Class I relationship. Skin changes were observed in 83.3% of patients with TMJ problems, whereas only 40.6% of patients without TMJ complaints experienced skin irritation. This difference was statistically significant (*P* = 0.004). The proportion of patients with skin irritation increased with a decrease in patient cooperation (Table 3).

Skin irritation occurred within 1 month after facemask delivery in more than half of the patients. In nine patients, it took more than 4 months until skin changes were observed (Table 4). Skin irritation occurred most frequently during the first month in both sex groups, although the distribution of the onset time in the two groups showed some differences. Patients with TMJ complaints most commonly experience skin erythema.

**Table 4:** The proportion of patients with skin irritation by onset time according to the variables.

Variable		Number of patients (percent)					x^2^ (*p*)
		< 1 month	< 2 months	< 3 months	> 4 months	Total	
Age (years)	6–7	12 (48.0%)	8 (28.0%)	4 (12.0%)	3 (12.0%)	27	9.788^†^ (0.108)
	8–9	22 (53.6%)	3 (10.7%)	9 (21.4%)	4 (14.3%)	38	
	> 10	5 (50.0%)	0 (0.0%)	5 (40.0%)	2 (10.0%)	12	
Gender	Female	24 (49.0%)	4 (8.2%)	16 (32.6%)	5 (10.2%)	49	9.064^†^ (0.026)
	Male	15 (53.6%)	7 (25.0%)	2 (7.1%)	4 (14.3%)	28	
Overjet (mm)	0 < OJ ≤ 4	4 (40.0%)	0 (0.0%)	4 (40.0%)	2 (20.0%)	10	3.815^†^ (0.232)
	OJ ≤ 0	35 (52.2%)	11 (16.4%)	14 (20.9%)	7 (10.4%)	67	
Overbite (mm)	0 < OB ≤ 4	29 (56.9%)	6 (11.8%)	9 (17.6%)	7 (13.7%)	51	7.185^†^ (0.251)
	OB > 4	3 (42.9%)	0 (0.0%)	3 (42.9%)	1 (14.3%)	7	
	OB ≤ 0	7 (36.8%)	5 (26.3%)	6 (31.6%)	1 (5.3%)	1	
ANB	Class I	23 (57.5%)	6 (15.0%)	8 (20.0%)	3 (4.7%)	40	3.187^†^ (0.804)
	Class II	3 (42.9%)	1 (14.3%)	2 (28.6%)	1 (14.3%)	7	
	Class III	13 (43.3%)	4 (13.3%)	8 (26.7%)	5 (16.7%)	30	
Wits	Class I	2 (50.0%)	1 (25.0%)	0 (0.0%)	1 (25.0%)	4	2.544^†^ (0.402)
	Class III	37 (50.7%)	10 (13.7%)	18 (24.7%)	8 (11.0%)	73	
TMJ complaints	Yes	6 (60.0%)	1 (10.0%)	2 (20.0%)	1 (10.5%)	10	0.422^†^ (1.000)
	No	33 (49.3%)	10 (14.9%)	16 (23.9%)	8 (11.9%)	67	
Patient cooperation	Good	8 (44.4%)	4 (22.2%)	4 (22.2%)	2 (2.1%)	18	2.777^†^ (0.863)
	Fair	15 (50.0%)	3 (10.0%)	7 (23.3%)	5 (16.7%)	30	
	Poor	16 (55.2%)	4 (13.8%)	7 (24.1%)	2 (6.9%)	29	
Chin angle	> Average	25 (55.6%)	7 (15.6%)	11 (24.4%)	2 (4.4%)	45	5.318^†^ (0.155)
	< Average	14 (43.8%)	4 (12.5%)	7 (21.9%)	7 (21.9)	32	
Total		39 (50.6%)	11 (14.3%)	18 (23.4%)	9 (11.7%)	77 (100%)	

*p < .05.

^†^Fisher’s exact test.

The analysis of 77 patients with skin irritation showed that erythema appeared in 67 patients (87.0%) and lacerations occurred in 10 patients (12.9%). Table 5 shows that TMJ complaints were significantly correlated with SII scores. In the group with TMJ complaints, the largest proportion of patients was categorized as SII 1 (83.3%), while most patients without TMJ symptoms showed SII 0 (59.4%).

**Table 5:** The proportion of patients by type of skin irritation according to the variables.

Variable		Number of patients (percent)				x^2^ (*p*)
		No change	Erythema	Laceration	Total	
Age (years)	6–7	23 (46.0%)	22 (44.0%)	5 (10.0%)	50	5.200^†^ (0.255)
	8–9	56 (59.6%)	35 (37.2%)	3 (3.2%)	94	
	> 10	21 (63.6%)	10 (30.3%)	2 (6.1%)	23	
Gender	Female	48 (49.5%)	41 (42.3%)	8 (8.2%)	97	5.392^†^ (0.072)
	Male	52 (65.0%)	26 (32.5%)	2 (2.5%)	80	
Overjet (mm)	0 < OJ ≤ 4	22 (68.8%)	10 (31.3%)	0 (0%)	32	5.223^†^ (.254)
	OJ > 4	1 (100%)	0 (0%)	0 (0%)	1	
	OJ ≤ 0	63 (53.4%)	47 (39.8%)	8 (6.8%)	118	
Overbite (mm)	0 < OB ≤ 4	69 (57.5%)	46 (38.3%)	5 (4.2%)	120	2.782^†^ (0.576)
	OB > 4	8 (53.3%)	5 (33.3%)	2 (13.3%)	15	
	OB ≤ 0	23 (54.8%)	16 (38.1%)	3 (7.1%)	42	
ANB	Class I	62 (61.2%)	36 (35.0%)	4 (3.9%)	103	4.547^†^ (0.302)
	Class II	6 (46.2%)	7 (53.8%)	0 (0.0%)	13	
	Class III	31 (50.8%)	24 (39.3%)	6 (9.8%)	61	
Wits	Class I	7 (63.6%)	3 (27.3%)	1 (9.1%)	11	1.158^†^ (0.571)
	Class III	93 (56.0%)	64 (38.6%)	9 (5.4%)	166	
TMJ complaints	Yes	2 (16.7%)	10 (83.3%)	0 (0.0%)	12	9.914 (0.005*)
	No	98 (59.4%)	57 (34.5%)	10 (6.1%)	165	
Patient cooperation	Good	32 (64.0%)	13 (26.0%)	5 (10.0%)	50	8.106^†^ (0.079)
	Fair	44 (59.5%)	27 (36.5%)	3 (4.1%)	74	
	Poor	24 (45.3%)	27 (50.9%)	2 (3.8%)	53	
Chin angle	> Average	51 (53.1%)	38 (39.6%)	7 (7.3%)	96	1.527^†^ (0.459)
	< Average	49 (60.5%)	29 (35.8%)	3 (3.7%)	81	
Total		100 (56.5%)	67 (37.9%)	10 (5.6%)	177 (100%)	

*p < .05.

^†^Fisher’s exact test.

Table 6 shows that, according to the logistic regression analysis, female patients were 2.2 times more likely to exhibit skin irritation than male patients. In the presence of TMJ complaints, the likelihood of experiencing skin problems was 21.5 times higher.

**Table 6:** Logistic regression model for skin irritation according to the variables.

Variable		Odds ratio	95% Confidence interval		*p* value
			Lower	Upper	
Age		0.792	0.601	1.044	0.098
Female		2.213	1.119	4.379	0.022*
Overjet		1.021	0.786	1.326	0.877
Overbite		1.235	0.987	1.543	0.064
ANB		0.873	0.683	1.117	0.281
Wits		1.141	0.955	1.363	0.145
TMJ complaints		21.481	3.435	134.333	0.001*
Patient cooperation	Good				0.085
	Fair	1.292	3.079	0.563	0.563
	Poor	2.570	6.351	0.041	0.041*
Chin angle		1.056	0.981	1.137	0.146

*p < .05.

## Discussion

Skin irritation is common in children undergoing orthodontic treatment with facemasks. Treatment success is highly dependent on patient cooperation and the duration of wearing the device; therefore, discomfort or pain related to skin changes can result in reduced treatment effects.

Our results showed that 43.5% of the patients experienced some form of skin reaction during treatment. No similar studies have investigated the prevalence of skin changes associated with facemask therapy. Most studies regarding adverse skin reactions during orthodontic treatment focus on nickel allergies caused by nickel-containing appliances^11–13^. Furthermore, there are data about reactions caused by extraoral appliances, while most studies are on the nickel component of headgear^14–18^.

While most of the adverse reactions to intraoral appliances are immunological reactions to allergens such as nickel, latex, or other components of orthodontic devices, skin changes in patients undergoing facemask therapy are often due to pressure on the skin or friction^19–21^. Although uncommon, allergic contact dermatitis associated with facemasks has been reported^22,23^. Allergic contact dermatitis is a pruritic, eczematous eruption that may be acute (blistering, weeping, and edema) or chronic (lichenified or scaly plaques)^24^. This reaction is typically well-demarcated and localized to the site of the skin that comes into contact with the allergen^24^. The skin changes observed in this study were not characteristic of allergic contact dermatitis (Fig. 1). Furthermore, they affected small areas in the lower part of the chin but not the forehead, which had contact with the facemask. Therefore, allergic contact dermatitis is excluded as a differential diagnosis for skin changes. There are several possible explanations for the affected skin in this study. These include irritant contact dermatitis or atopic diathesis, in correlation with epidermal barrier dysfunction.

Irritant contact dermatitis is a localized, non-immunological cutaneous inflammatory reaction with polymorphous clinical features. Erythema, scaling, edema, vesiculation, and erosion can occur in acute cases. In chronic cases, lichenification, hyperkeratosis, and fissures are observed. Frictional contact dermatitis is a subtype of irritant contact dermatitis resulting from repeated low-grade frictional trauma, such as wearing a facemask, as in this study^24,25^.

Atopic diathesis, encompassing atopic dermatitis, allergic rhino-conjunctivitis, food allergy, eosinophilic esophagitis, and asthma, is commonly associated with epidermal barrier dysfunction^26^. The skin barrier function resides primarily in the stratum corneum of the epidermis^27^. The epidermal barrier function can be measured using transepidermal water loss (TEWL) and stratum corneum hydration (SCH)^26^. TEWL evaluates the diffusion of condensed water through the stratum corneum, and a greater TEWL is often associated with skin barrier impairment^26,28^. SCH describes the water content of the stratum corneum, and a lower value is associated with skin barrier dysfunction^26^. A number of studies reported that TEWL decreases with age, implicating a weaker skin barrier in younger ages. This may explain the increased frequency of skin changes observed in the younger age groups in this study. However, in children with eczema, a decrease in SCH is reported regardless of sex and age, while no significant changes in SCH could be observed in children without eczema^29^. These findings suggest that the difference in skin condition among participants in our study might depend on the presence of atopic diathesis.

We found that skin irritations occur more often in female than male patients. The epidermis is the outermost layer of the skin, providing a protective barrier against mechanical stimuli or potentially harmful environmental agents and regulating the loss of water and electrolytes^30^. Depending on the thickness of the epidermis, the protective and regulatory functions of the skin can vary. Several studies have reported that the epidermis is thicker in men than in women^30–33^. This suggests that male skin might have a better protective function of the epidermis against mechanical stress and frictional forces than female skin, which is consistent with the results of our study.

In this study, malocclusion severity and chin angle were not significantly correlated with skin irritation, although there was a tendency toward increased skin problems in patients with more severe malocclusion. However, the presence of complaints in the TMJ was statistically significant. From these results, it can be inferred that skin irritation is more closely associated with the force applied than with individual anatomical variations. The facemask appliance is fitted to sit passively on the forehead and chin, while the protraction force is generated by the elastics. The force is adjusted by the elastics’ size and the crossbar position. Specifically, the chin position related to the prognathic mandible does not usually affect the total force applied, which can be measured and calibrated using a force gauge. However, the amount of stress exerted on the chin and TMJ areas may vary depending on the appliance’s design and the direction of the force applied^7,34,35^. Skin irritation and TMJ complaints may both result from the excessive force delivered by the appliance to the TMJ and chin areas^36^. On the other hand, skin irritation can also occur secondary to TMJ pain. When the mandible is forced posteriorly, the condyle can press against the sensitive retrodiscal bilaminar zone, causing pain or discomfort^37,38^. For pain relief, the patient may develop a habit of mandibular thrusting, which increases the pressure on the chin area and the risk of skin irritation. However, the cause-and-effect relationship between the occurrence of skin irritation and TMJ complaints is unclear and requires further investigation in future studies.

The gradual development of 3D technology has opened new possibilities for the production of orthodontic devices. With the help of a 3D face scanner, the 3D-printed orthodontic facemask can be fully customized. Such customization can improve device fit and patient comfort^39^. Furthermore, it can reduce skin irritation if the chin cup is modified in shape and material to fit the individual anatomy and skin type. However, the need for additional equipment and the high costs of manufacturing customized facemasks make their application to every patient difficult. As an alternative to full customization, the chin cup component of the prefabricated facemask can be modified to prevent skin irritation. Chair-side customization using polyvinyl siloxane for a better fit and stability has been suggested for better stress distribution in the chin area^40^. Furthermore, lining the internal surface of the chin cup with skin-protecting bandaging materials such as cotton cloth, hydrocolloid, or foam could help protect the skin and prevent skin irritation. In addition, the topical application of zinc paste can also enhance the skin barrier to a certain degree.

There are limitations to this study. This study did not include a control group, and the data were collected retrospectively. Due to the retrospective nature of the study and for ethical reasons, possible effects of confounders, such as the self-application of topicals or the use of cloths or pads by the patients or their guardians for the prevention or reduction of skin problems, were not strictly controlled. Furthermore, we did not observe any apparent season-dependent effects, such as sweating in the summer or skin dehydration during very cold months, in our patients. The sample size of some subgroups was relatively small, while the size of the study population was sufficient. The results of this study should be interpreted with caution regarding cause-and-effect relationships. Despite these limitations, this study is the first to systematically investigate the skin irritation associated with orthodontic facemasks.

## Conclusion

This study confirmed that skin irritation is a frequent complication of facemask therapy, affecting nearly half (43.5%) of the patients. Skin changes were observed more frequently in female patients than in male patients, as well as in patients who developed TMJ problems during treatment. To minimize skin irritation associated with facemasks, patients and their parents should be informed of possible skin reactions and provided with sufficient guidelines regarding the correct use of facemasks and management in case of skin problems during treatment.

## Data Availability

The data underlying this article cannot be publicly shared to protect the privacy of the individuals participating in the study. The data will be shared at a reasonable request from the corresponding author.
